# Mental health status of adolescents in-home quarantine: a multi-region, cross-sectional study during COVID-19 pandemic in Bangladesh

**DOI:** 10.1186/s40359-022-00819-3

**Published:** 2022-05-05

**Authors:** Sadia Afrin, Sarker Mohammad Nasrullah, Koustuv Dalal, Zarrin Tasnim, Md. Shadly Benzadid, Farhin Humayra, K. M. Saif-Ur-Rahman, Mohammad Delwer Hossain Hawlader

**Affiliations:** 1 Department of Public Health, North South University, Dhaka, 1229 Bangladesh; 2 Public Health Professional Development Society (PPDS), Dhaka, 1215 Bangladesh; 3 Division of Public Health Science, School of Health Sciences, Mid Sweden University, Sundsvall, Sweden; 4 Augmedix, Panthapath, Dhaka, Bangladesh; 5 Dept. of Medicine, Shaheed Suhrawardy Medical College and Hospital, Dhaka, 1207 Bangladesh; 6 Health System and Population Studies Division, ICDDRB, Dhaka, 1212 Bangladesh

**Keywords:** Mental health, Depression, Anxiety, Stress, Adolescents, Home quarantine, Lockdown, Pandemic, COVID-19, Bangladesh

## Abstract

**Background:**

The population's mental and physical health worldwide are currently at risk due to the coronavirus pandemic. We evaluated the mental health status of the adolescents trapped indoors because of the precautionary restrictions and prolonged closure of the educational institutions.

**Method:**

A cross-sectional study was conducted on adolescents from multiple urban and semi-urban areas of Bangladesh from 22 January to 3 February 2021. A self-reported online questionnaire containing questions regarding sociodemographic factors, home quarantine-related factors and mental health symptoms was distributed to collect data. Descriptive analysis, bivariate and multivariable logistic regressions were performed to measure the association of the variables. Cronbach's alpha was estimated to present the internal consistency of the scales.

**Results:**

A total of 322 adolescents (aged 12–19) with a mean age of 16.00 years (SD = 1.84) responded to the invitation. 54.97% (n = 177) of them were male, and the participants were predominantly urban residents (87.27%, n = 281). We observed varying degrees of depression in 67.08%, anxiety in 49.38% and stress in 40.68% of the participants according to DASS-21. Age, sex, education, mother's occupation, total monthly income, playing sports, doing household chores, going out of home, watching television, using the internet, attending online classes, changing food habits, and communicating with friends had a positive significant association with mental health burdens.

**Conclusion:**

Home quarantine has a noticeable adverse impact on the mental health of teenagers. Psychological evaluations and counselling via online and offline programs are essential to improve adolescents' declining mental health conditions.

## Background

SARS-CoV-2 has been declared a global public health emergency by WHO, with around 120 million confirmed cases and 2.6 million deaths across nearly 200 countries [1–3]. To impede the transmission of COVID-19, countries worldwide have adopted 'social distancing' strategies by locking regions down, online schooling, work from home policy, keeping suspected and confirmed cases under home quarantine [4]. Constant health concerns and strict alteration of lifestyle have had consequential detrimental effects on the mental health as well as the physical health of people [5, 6]. Adolescence is a delicate stage and crucial for social, mental and cognitive development [7]. Coping with the current situation is a more complicated challenge for adolescents, and it has a substantial impact on their mental health [8, 9]. They are burdened with increased mental health issues due to home confinement during the pandemic [9–13]. The world is already facing increasing cases of mental illness in the young population and in the USA, the number of students looking for mental health counselling is rising alarmingly [14]. A study in China showed that 44% of 12–18-year-olds showed negative behaviour [15]. The coronavirus crisis further deteriorated these situations [16]. A study in Italy and Spain showed that the pandemic had exacerbated psychiatric problems such as behavioural difficulties, abandonment, and low spirits in adolescents [17]. Adolescents make up about 10.2% of Bangladesh's overall population (16.4 million; 8.4 million boys and 8.0 million girls). According to studies, Bangladeshi adolescents affected from anxiety, loneliness, a lack of close relationships, bullying, substance usage, and smoking [18]. A research study before the lockdown revealed that adolescent girls were more prone to depression because of customs and social stigmas in Bangladesh. According to another study conducted during the pandemic, around 18.1% of Bangladeshi university students were distressed [19]. Moreover, physical abuse was on the rise, affecting children and adolescents' mental and psychological development [20]. Mental health has not received much emphasis in the developing countries like Bangladesh.

Despite previous studies on the mental health status of adolescents during this pandemic, a gap in the literature remains regarding the association between the factors related to their daily lives in lockdown and mental health. We seek to bridge this gap by evaluating the impact of various factors in-home quarantine on the mental health status of the adolescents of multiple urban and semi-urban regions of Bangladesh during the pandemic. We aim to assess the distribution of different mental health burdens among the study population and look for the associated factors regarding their lives in-home quarantine.

## Methods

### Study design and participants

A descriptive, cross-sectional study was conducted on adolescents from several urban and semi-urban areas in Dhaka, Chittagong, Sirajganj and Kushtia in Bangladesh. Urban and semi-urban areas refer to City Corporation and City Council (Pourashabha). Based on the socio-economic circumstances in our country, we consider lower, lower-middle, and upper-middle-class people to be their parents. The inclusion criteria were: being a student of classes 6 to 10 and college, never diagnosed with any mental disorder by a doctor and willing to participate in the research. On the other hand, those above 19 or below 12 years of age were excluded. The study comprised 340 respondents (143 from Dhaka, 97 from Sirajganj, 52 from Kushtia and 48 from Chittagong) who submitted answers; 322 participants were selected for the final analysis after omitting the incomplete responses.

### Data collection procedure

**Fig. 1:**
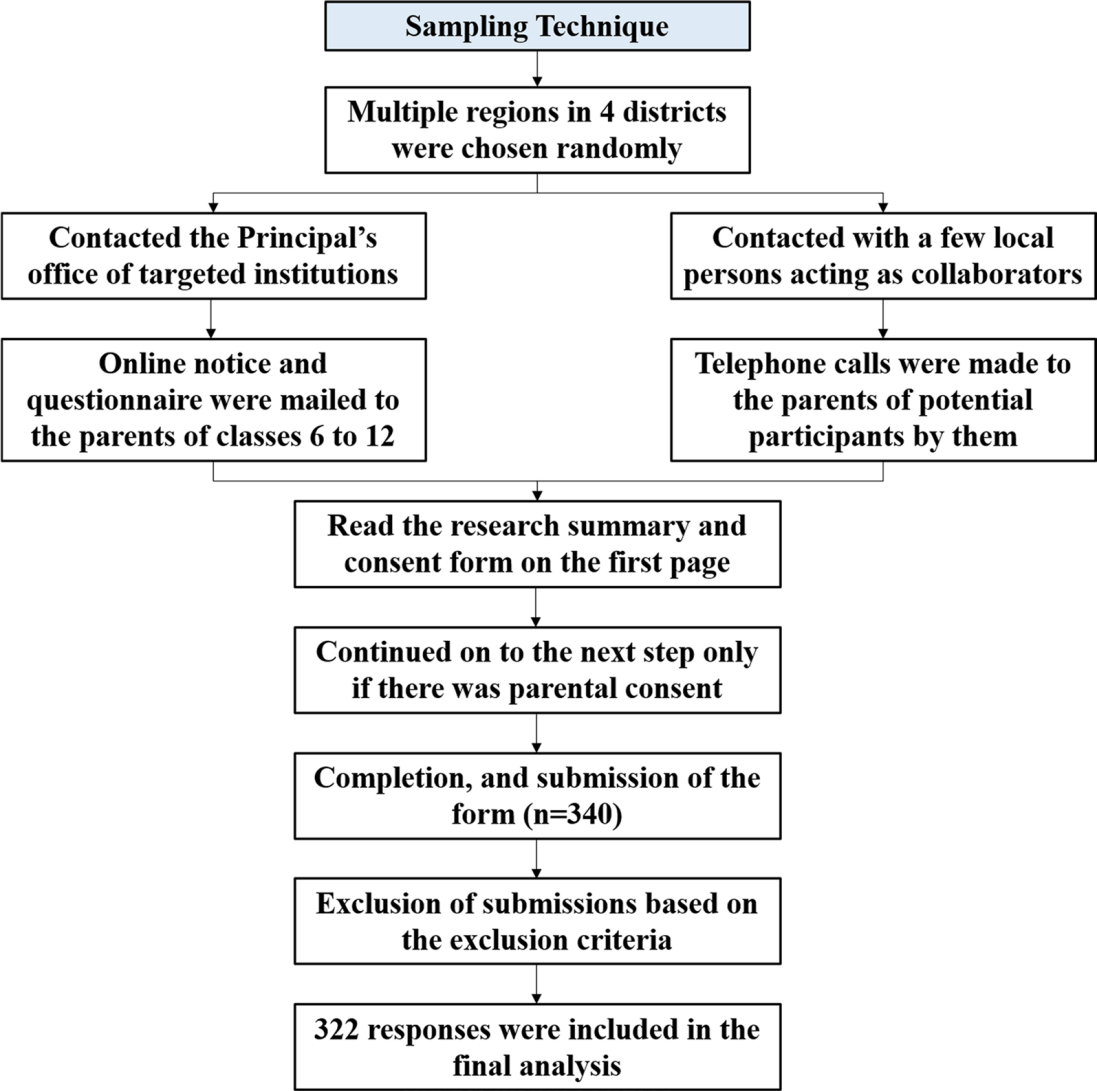
Sampling technique of the study

The Government of Bangladesh had declared the closure of all educational institutions on and from 18 March 2020. Till February 2022, all the educational institutions have been closed [21]. We collected our data in two ways- via google form and telephone. The ongoing crisis did not permit us to conduct face-to-face interviews on the premises of respective educational institutions. Moreover, we avoided visiting people's residences due to health concerns. Following prior appointments, online surveys and telephone interviews were carried out from 22 January to 3 February 2021. We collaborated with a number of local persons from each region. These collaborators were school teachers who had access to a pool of adolescent students and their parents by their profession. They kindly helped distribute the questionnaire online and made phone calls to the guardians of the students where necessary. Around one-third of the urban participants were from English medium schools. Only these participants got the original English questionnaire. The rest got the translated questionnaire or took part in the interviews in Bengali. A mixed sampling method was adopted for primary data collection (Fig. 1).

A pilot study was carried out in a sample of 40 subjects before the final study. The questionnaire contained four parts: (1) title, summary and consent form, (2) sociodemographic information, (3) home quarantine-related factors and (4) DASS-21 items. The respondents had to fill out the self-reporting questionnaire after obtaining consent from their parents and had the liberty of dropping out of the survey at any time.

### Mental health assessment of adolescents

We used the depression-anxiety-stress scale-21 (DASS-21) to evaluate adolescents' frequency of mental health issues [22]. The scale was also endorsed in the native language Bangla [23]. The scale contains 21 items divided equally, with seven items into three subscales of stress, anxiety and depression. The total score from each subsection can range from average to highly severe. The students responded to the items on a 4-point Likert scale (0 = never a problem, 1 = sometimes a problem, 2 = often a problem, and 3 = almost always a problem). Example items include "I found it hard to wind down" (stress), "I was aware of dryness of my mouth" (anxiety), "I couldn't seem to experience any positive feeling at all" (depression). The adolescents had to reply to 21 items on a 4-point Likert scale. The outcome varied from 'normal' to highly severe based on total points in the case of each mental health problem.

### Statistical analysis

Stata 16 (Stata Corp LLC, College Station, TX, USA) was used to execute the data analysis. Descriptive statistics were used to express the participants' sociodemographic characteristics and home quarantine-related variables. Sociodemographic factors consisted of age, sex, place of residence, level of education of the participants and parents, number of members in the family, parents' occupation and total monthly income of the family. Home quarantine related variables were: whether involved in extra-academic activities like outdoor and indoor games, hobby and household chores, duration of watching television, studying, sleep and internet use, the purpose of internet use, any change in food habit or not, the frequency of communication with friends and whether the participant wished for the schools/colleges to reopen soon or not. At first, bivariate logistic regression was used to calculate the unadjusted odds ratios. Significantly correlated variables were then included in the multiple logistic regression model to estimate adjusted odds ratios as measures of association with psychological estimates. Statistical significance was set at a *p* value of less than 0.05 and 95% confidence intervals were mentioned. The reliability of the scales was presented through Cronbach's alpha scores. An alpha score of 0.70 or above was considered to be acceptable.

### Ethical issues

The research proposal was reviewed and approved by the Ethics Review Committee (ERC)/Institutional Review Board (IRB) of North South University (2020/OR-NSU/IRB-No.0801). All methods were performed in accordance with the relevant guidelines and regulations. Informed written consents were obtained.

In most cases, we contacted the principal's office of a targeted school/college to seek permission to conduct the study. Upon formal clearance, online notices or emails bearing the official seal were issued to the parents along with the questionnaire. The parents then read the research summary and consent on the first page of the online questionnaire. They were requested to continue to the next step only if they wished that their children participate in the study. In places where we could not approach the office of any academic institution, we had to rely entirely on the research collaborators to ensure that the parents/legal guardians read the consent form to secure that the inclusion criteria were met.

## Results

### Descriptive statistics

From 340 respondents, the final analysis included 322 adolescents (Table 1) with a mean age of 16.00 years (SD = 1.84). 54.97% (n = 177) of them were male, and predominantly urban residents (87.27%, n = 281). The majority of the students were from 6 to 10th grade (66.46%, n = 214), while the rest were in college. 80.43% (n = 259) and 64.60% (n = 208) of the respondents' fathers and mothers were university graduates or above, respectively. Fathers were mostly in public or private services (55.28%, n = 178). On the other hand, mothers were mostly homemakers (68.63%, n = 221). (44.72%, n = 144) families had a total income of 20,000 to 40,000 BDT per month.

**Table 1:** Sociodemographic characteristics of the participants

Variables	Frequency (n/322)	Percentage (%)
*Age (in years)* *Mean ± SD*, 16.00 ± 1.84		
12–14	70	21.74
15–17	171	53.11
18–20	81	25.16
*Gender*		
Male	177	54.97
Female	145	45.03
*Place of residence*		
Urban	281	87.27
Sub-urban or rural	41	12.73
*Level of education*		
6th to 10th grade	214	66.46
College	108	33.54
*Members in family*		
≤ 4	214	66.46
> 4	108	33.54
*Father’s highest education*		
School	23	7.14
College	40	12.42
Graduate or above	259	80.43
*Mother’s highest education*		
School	45	13.98
College	69	21.43
Graduate or above	208	64.60
*Father’s occupation*		
Retired or unemployed	14	4.35
Public or private service	178	55.28
Business	80	24.84
Agriculture	3	0.93
Others	47	14.60
*Mother’s occupation*		
Retired	1	0.31
Public or private service	74	22.98
Business	9	2.80
Home-maker	220	68.32
Others	18	5.59
*Total family income (in BDT)*		
< 20,000	78	24.22
20,000–40,000	144	44.72
40,001–100,000	26	8.07
> 100,000	74	22.98

Table 2 presents the characteristics related to the lifestyle and behaviour of adolescents in-home quarantine. The students were more inclined towards playing games indoors (66.15%, n = 213). However, 25.16% (n = 81) reported playing outside the house. About one-third of the respondents (34.16%, n = 110) claimed that they did not watch television at all during quarantine. 37.58% (n = 121) of the students did extensive studying for more than 3 h each day. Interestingly, 5.28% (n = 17) stated that they did not study during the lockdown. A large part of the respondents (63.66%, n = 205) had the routine of using the internet for more than 3 h every day. A considerable part of the participants (77.33%, n = 249) spent time on their hobbies and 80.75% (n = 260) did daily household chores. It was observed that 58.39% (n = 188) of the students slept for at least 6 h or more. Whereas a small portion (1.86%, n = 6) did not sleep at all at night. 58.70% (n = 189) students reported changing their food habits during quarantine. Almost half of the adolescents (50.31%, n = 162) stated less communication with their friends than usual during this pandemic. Only 11.18% (n = 36) of the students declared that they never went out or very rarely left home. Many students (84.47%, n = 172) were attending online classes from home. Another curious finding was that 41.30% (n = 133) of the adolescents did not want their schools/colleges to reopen.

**Table 2:** Home quarantine related characteristics of the participants

Variables	Frequency (n/322)	Percentage (%)
*Plays outdoor games*		
Yes	81	25.16
No	241	74.84
*Plays indoor games*		
Yes	213	66.15
No	109	33.85
*Duration of watching television*		
Not at all	110	34.16
Less than an hour	103	31.99
1 to 3 h	84	26.09
More than 3 h	25	7.76
*Duration of studying*		
Not at all	17	5.28
Less than an hour	73	22.67
1 to 3 h	111	34.47
More than 3 h	121	37.58
*Duration of internet use*		
Less than an hour	23	7.15
1 to 3 h	94	29.19
More than 3 h	205	63.66
*Uses internet for social networking*		
Yes	274	85.09
No	48	14.91
*Uses internet for online gaming*		
Yes	151	46.89
No	171	53.11
*Uses internet for other entertainments*		
Yes	270	83.85
No	52	16.15
*Spends time in any hobby*		
Yes	249	77.33
No	73	22.67
*Does household chores*		
Yes	260	80.75
No	62	19.25
*Duration of sleep at night*		
No sleep	6	1.86
Inadequate sleep	128	39.75
Adequate sleep	188	58.39
*Change in food habit*		
No change	133	41.30
Eats more or less than usual	189	58.70
*Communication with friends*		
As usual	104	32.30
Less than usual	162	50.31
More than usual	56	17.39
*Frequency of going out*		
Never or very rarely	36	11.18
Sometimes	277	86.02
Frequently	9	2.80
*Attends online classes*		
Yes	172	84.47
No	50	15.53
*Wants school/college to reopen*		
Yes	189	58.70
No	133	41.30

**Fig. 2:**
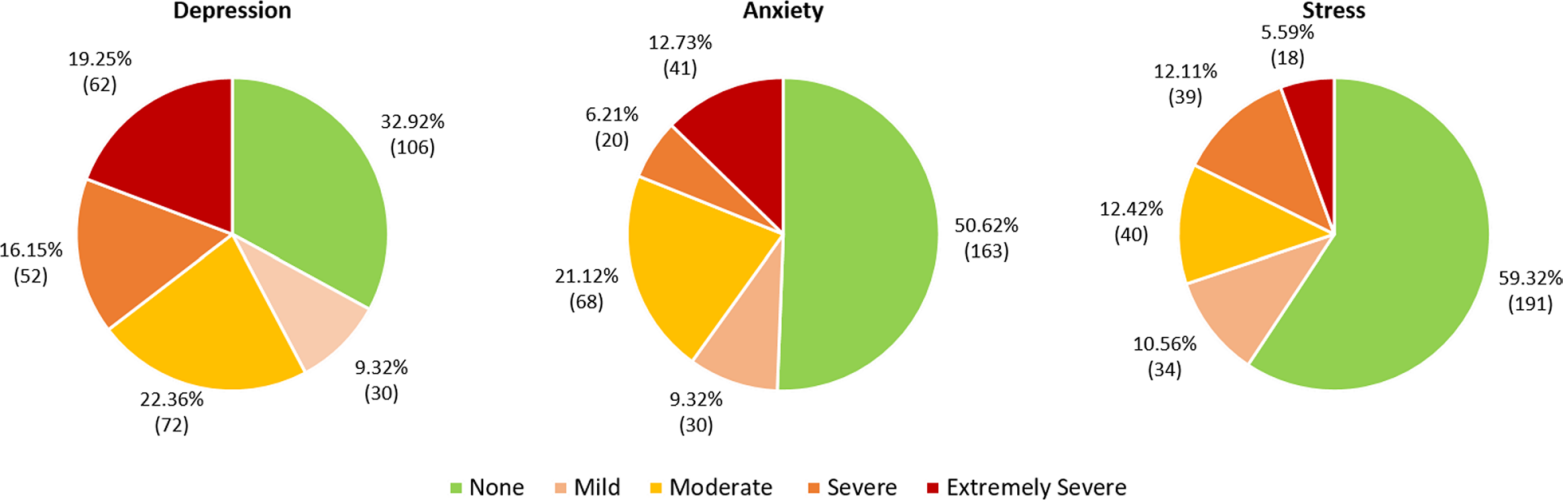
Prevalence of depression, anxiety and stress based on DASS-21

Figure 2 displays the prevalence of mental health burdens in adolescents. Among the 322 participants who fully completed the questionnaire, some levels of depression, anxiety and stress were observed in 216 (67.08%), 159 (49.38%) and 131 (40.68%) adolescents. Severe symptoms of depression were found in 16.15% (n = 52). 6.21% (n = 20) and 12.11% (n = 39) had severe anxiety and stress respectively. An extremely severe level of symptoms of depression, anxiety and stress was detected in 62 (19.25%), 41 (12.73%) and 18 (5.59%) of the study participants, respectively.

Table 3 summarizes the scores of DASS-21 scale along with Cronbach's alpha scores. It is noticeable that the total score of the seven items from each domain ranges from 0 to 21. The depression and the stress scale had alpha scores above 0.85, showing excellent internal consistency. The reliability score of the anxiety scale (0.79) was also within an acceptable range.

**Table 3:** Descriptive statistics and Cronbach’s alpha of the scales (n = 322)

Scale	Mean	SD	Range	Cronbach’s alpha
Depression	8.25	5.48	0–21	0.88
Anxiety	4.42	3.86	0–21	0.79
Stress	7.25	5.05	0–21	0.86

### Association with depression

The following variables were significantly associated with depression in bivariate analysis (Tables 4, 5): age over 14 years of age (15–17 years: OR = 2.30, 95% CI = 1.30, 4.06; *p* value = 0.004) (18–19 years: OR = 5.86, 95% CI = 2.75, 12.49; *p* value = < 0.001), studying in college (OR = 3.81, 95% CI = 2.12, 6.84; *p* value =  < 0.001), using internet for more than 3 h (OR = 2.51, 95% CI = 1.05, 6.01; *p* value = 0.038), eating more or less than usual (OR = 3.45, 95% CI = 2.12, 5.60; *p* value = < 0.001) and having less communication with friends than usual (OR = 2.34, 95% CI = 1.38, 3.95; *p* value = 0.001), being a male (OR = 0.47, 95% CI = 0.29, 0.77; *p* value = 0.003), playing outdoors (OR = 0.51, 95% CI = 0.30, 0.85; *p* value = 0.011) and attending online classes (OR = 0.45, 95% CI = 0.21, 0.95; *p* value = 0.038).

**Table 4:** Association of mental health burdens with sociodemographic characteristics

Variables	Depression		Anxiety		Stress	
	OR (95% CI)	AOR (95% CI)	OR (95% CI)	AOR (95% CI)	OR (95% CI)	AOR (95% CI)
*Age group (years)*						
12–14	Reference	Reference	Reference	Reference	Reference	Reference
15–17	2.30 (1.30–4.06)**	1.64 (0.82–3.24)	1.09 (0.62–1.91)	1.28 (0.69–2.37)	1.02 (0.56–1.84)	0.85 (0.40–1.80)
18–20	5.86 (2.75–12.49)***	0.98 (0.23–4.13)	2.39 (1.24–4.60)**	3.08 (1.03–9.23)*	3.47 (1.77–6.80)***	2.54 (0.71–9.03)
*Gender*						
Female	Reference	Reference	Reference	Reference	Reference	Reference
Male	0.47 (0.29–0.77)**	0.57 (0.32–1.02)	0.53 (0.34–0.83)**	0.59 (0.36–0.96)*	0.34 (0.21–0.55)***	0.47 (0.26–0.82)**
*Place of residence*						
Sub-urban or rural	Reference		Reference		Reference	
Urban	0.53 (0.24–1.16)		0.91 (0.47–1.77)		0.76 (0.39–1.48)	
*Level of education*						
6th to 10th grade	Reference	Reference	Reference	Reference	Reference	Reference
College	3.81 (2.12–6.84)***	3.88 (1.20–12.51)*	1.82 (1.13–2.91)*	0.74 (0.31–1.75)	2.66 (1.65–4.29)***	0.89 (0.33–2.37)
*Members in family*						
≤ 4	Reference		Reference		Reference	
> 4	0.85 (0.52–1.39)		0.87 (0.55–1.39)		1.12 (0.70–1.80)	
*Father’s highest education*						
School	Reference		Reference		Reference	
College	3.07 (0.99–9.53)		1.63 (0.58–4.60)		1.71 (0.60–4.87)	
Graduate or above	1.49 (0.63–3.54)		1.00 (0.42–2.35)		0.99 (0.41–2.38)	
*Mother’s highest education*						
School	Reference		Reference		Reference	
College	1.46 (0.61–3.46)		0.95 (0.45–2.03)		1.07 (0.50–2.28)	
Graduate or above	0.67 (0.33–1.36)		1.04 (0.54–1.99)		0.57 (0.30–1.10)	
*Father’s occupation*						
Retired or unemployed	Reference		Reference		Reference	
Public or private service	0.30 (0.06–1.40)		1.64 (0.53–5.10)		0.58 (0.19–1.75)	
Business	0.27 (0.05–1.32)		1.54 (0.47–5.03)		0.77 (0.24–2.42)	
Agriculture	1		0.9 (0.06–12.58)		0.5 (0.03–6.86)	
Others	0.54 (0.10–2.81)		3.48 (1.00–12.15)		0.88 (0.26–2.90)	
*Mother’s occupation*						
Retired	Reference		Reference	Reference	Reference	
Public or private service	2.31 (0.79–6.72)		3.41 (1.10–10.55)*	3.18 (0.98–10.31)	2.09 (0.67–6.46)	
Business	2.79 (0.45–17.38)		5.19 (0.92–29.26)	4.69 (0.78–28.02)	3.25 (0.61–17.28)	
Home-maker	1.51 (0.57–3.99)		2.37 (0.81–6.88)	2.47 (0.81–7.46)	1.73 (0.59–5.03)	
Others	1		1	1	1	
*Total family income (BDT)*						
< 20,000	Reference		Reference		Reference	Reference
20,000–40,000	0.93 (0.34–2.54)		1 (0.41–2.42)		0.56 (0.23–1.38)	0.49 (0.15–1.52)
40,001–100,000	0.73 (0.28–1.87)		1.18 (0.51–2.72)		0.68 (0.29–1.58)	0.63 (0.21–1.86)
> 100,000	0.57 (0.21–1.52)		0.64 (0.26–1.58)		0.36 (0.14–0.90)*	0.34 (0.10–1.13)

Only the variables with significant *p* value from bivariate analyses were included in the adjusted analysis

*OR* odds ratio, *AOR* adjusted odds ratio, *CI* confidence interval

*p* value: < 0.05*; < 0.01**; < 0.001***

**Table 5:** Association of mental health burdens with home quarantine related characteristics

Variables	Depression		Anxiety		Stress	
	OR (95% CI)	AOR (95% CI)	OR (95% CI)	AOR (95% CI)	OR (95% CI)	AOR (95% CI)
*Plays outdoor games*						
No	Reference	Reference	Reference		Reference	Reference
Yes	0.51 (0.30–0.85)*	0.62 (0.34–1.15)	0.82 (0.49–1.35)		0.48 (0.28–0.84)*	0.58 (0.30–1.13)
*Plays indoor games*						
No	Reference		Reference		Reference	
Yes	0.64 (0.38–1.06)		0.67 (0.42–1.06)		0.68 (0.42–1.09)	
*Duration of watching television*						
Not at all	Reference		Reference		Reference	Reference
Less than an hour	1.29 (0.36–4.58)		0.93 (0.54–1.60)		0.45 (0.26–0.80)***	0.42 (0.21–0.82)*
1 to 3 h	0.56 (0.17–1.86)		1.30 (0.73–2.30)		0.96 (0.54–1.70)	0.89 (0.45–1.77)
More than 3 h	0.45 (0.13–1.46)		0.99 (0.41–2.36)		0.74 (0.30–1.79)	1.29 (0.44–3.77)
*Duration of studying*						
Not at all	Reference		Reference		Reference	
Less than an hour	1.29 (0.36–4.58)		0.96 (0.33–2.77)		1.01 (0.35–2.93)	
1 to 3 h	0.56 (0.17–1.86)		0.81 (0.29–2.25)		0.58 (0.20–1.62)	
More than 3 h	0.45 (.13–1.46)		0.84 (0.30–2.33)		0.42 (0.15–1.17)	
*Duration of internet use*						
Less than an hour	Reference	Reference	Reference		Reference	
1 to 3 h	2.11 (0.84–5.31)	1.22 (0.42–3.52)	1.14 (0.45–2.86)		1.41 (0.53–3.78)	
More than 3 h	2.51 (1.05–6.01)*	1.18 (0.43–3.23)	1.36 (0.57–3.25)		1.71 (0.67–4.35)	
*Uses internet for social networking*						
No	Reference	Reference	Reference		Reference	Reference
Yes	3.17 (1.69–5.94)***	1.97 (0.91–4.26)	1.59 (0.85–2.97)		2.03 (1.03–4.01)*	2.23 (0.92–5.40)
*Uses internet for online gaming*						
No	Reference		Reference		Reference	
Yes	0.74 (0.46–1.18)		0.79 (0.51–1.23)		0.64 (0.41–1.01)	
*Uses internet for other entertainments*						
No	Reference	Reference	Reference		Reference	
Yes	2.37 (1.29–4.34)**	2.31 (1.08–4.93)*	1.40 (0.77–2.55)		1.66 (0.88–3.15)	
*Spends time on a hobby*						
No	Reference		Reference		Reference	
Yes	0.77 (0.44–1.37)		0.75 (0.44–1.27)		0.73 (0.43–1.23)	
*Does household chores*						
No	Reference		Reference		Reference	Reference
Yes	0.72 (0.39–1.34)		1.23 (0.70–2.15)		0.41 (0.23–0.73)**	0.53 (0.27–1.05)
*Duration of sleep at night*						
No sleep	Reference		Reference		Reference	
Inadequate sleep	0.51 (.05–4.52)		0.56 0(.10–3.20)		0.40 (0.07–2.27)	
Adequate sleep	0.34 (.03–3.01)		0.43 (0.07–2.40)		0.29 (0.05–1.66)	
*Change in food habit*						
No change	Reference	Reference	Reference	Reference	Reference	Reference
Eats more or less than usual	3.45 (2.12–5.60)***	3.23 (1.86–5.61)***	1.82 (1.16–2.86)**	1.54 (0.95–2.50)	2.72 (1.68–4.38)***	2.52(1.42–4.48)**
*Communication with friends*						
As usual	Reference	Reference	Reference	Reference	Reference	Reference
Less than usual	2.34 (1.38–3.95)***	2.54 (1.38–4.69)**	2.28 (1.37–3.78)***	2.34 (1.37–4.01)**	2.52 (1.48–4.28)**	2.59 (1.37–4.90)**
More than usual	1.54 (.78–3.03)	1.82 (.84–3.91)	1.86 (0.96–3.60)	2.15 (1.07–4.33)*	2.18 (1.10–4.32)*	2.83 (1.26–6.34)*
*Frequency of going out*						
Never or very rarely	Reference		Reference		Reference	Reference
Sometimes	0.91 (0.43–1.94)		0.68 (0.34–1.39)		0.40 (0.19–0.81)*	0.40 (0.106-.97)*
Frequently	0.35 (0.07–1.56)		0.20 0(.03–1.12)		0.18 (0.03–1.00)	0.17 (0.02–1.45)
*Attends online classes*						
No	Reference	Reference	Reference		Reference	Reference
Yes	0.45 (0.21–0.95)*	0.63 (0.27–1.47)	0.66 (0.36–1.21)		0.47 (0.26–0.88)*	0.68 (0.32–1.41)
*Wants school/college to reopen*						
No	Reference		Reference		Reference	
Yes	0.75 (0.46–1.21)		0.93 (0.59–1.45)		0.77 (0.49–1.21)	

Only the variables with significant *p* value from bivariate analyses were included in the adjusted analysis

*OR* odds ratio, *AOR* adjusted odds ratio, *CI* confidence interval

*p* value: < 0.05*; < 0.01**; < 0.001***

However, among the variables stated above, only four were found to be significantly associated with depressive symptoms in the adolescents after adjusting other factors (Tables 4, 5): studying in college (AOR = 3.88, 95% CI = 1.20, 12.51; *p* value = 0.023), using the internet for entertainments such as watching movies, listening to songs etc. (AOR = 2.31, 95% CI = 1.08, 4.93; *p* value = 0.03), any change in food habit (AOR = 3.23, 95% CI = 1.86, 5.61; *p* value = < 0.001), and having less contact with friends than usual (AOR = 2.54, 95% CI = 1.38, 4.69; *p* value = 0.003).

### Association with anxiety

Unadjusted analysis of the variables revealed significant association between symptoms of anxiety and the following factors (Tables 4, 5): age (18–19 years: OR = 2.39, 95% CI = 1.24, 4.61; *p* value = 0.009), male sex (OR = 0.53, 95% CI = 0.34, 0.83; *p* value = 0.006), studying in college (OR = 1.82, 95% CI = 1.13, 2.91; *p* value = 0.012), mother's occupation (public/private services: OR = 3.41, 95% CI = 1.10, 10.55; *p* value = 0.033), change in food habit (OR = 1.82, 95% CI = 1.16, 2.86; *p* value = 0.008) and less communication with friends (OR = 2.28, 95% CI = 1.37, 3.78; *p* value = 0.001).

Adjusted analysis revealed significant association between anxiety and the following variables in the adolescents (Tables 4, 5): age group of 18–20 years (AOR = 3.08, 95% CI = 1.03, 9.23; *p* value = 0.044), male sex (AOR = 0.59, 95% CI = 0.36, 0.96; *p* value = 0.036), having less contact with friends than usual (AOR = 2.34, 95% CI = 1.37, 4.01; *p* value = 0.002) and also having more communication with friends than usual (AOR = 2.15, 95% CI = 1.07, 4.33; *p* value = 0.031).

### Association with stress

In unadjusted analysis, the following variables were observed to be significantly associated with stress among the participants (Tables 4, 5): age (18–19 years: OR = 3.47, 95% CI = 1.77, 6.80; *p* value = < 0.001), male sex (OR = 0.34, 95% CI = 0.21, 0.55; *p* value = < 0.001), being a college student (OR = 2.66, 95% CI = 1.65, 4.29; *p* value = < 0.001), monthly family income of more than 1,00,000 BDT (OR = 0.36, 95% = 0.14, 0.90; *p* value = 0.03), watching television for less than an hour (OR = 0.45, 95% CI = 0.26, 0.80; *p* value = 0.007), using social platforms online (OR = 2.03, 95% CI = 1.03, 4.01; *p* value = 0.04), playing sports outdoor (OR = 0.48, 95% CI = 0.28, 0.84; *p* value = 0.01), doing daily household chores (OR = 0.41, 95% CI = 0.23, 0.73; *p* value = 0.002), eating more or less than usual (OR = 2.72, 95% CI = 1.68, 4.38; *p* value =  < 0.001), and having less communication with friends (OR = 2.18, 95% CI = 1.10, 4.32; *p* value = 0.024), going out of the house (OR = 0.40, 95% CI = 0.19, 0.81; *p* value = 0.012) and attending online classes (OR = 0.47, 95% CI = 0.26, 0.88; *p* value = 0.018).

In multiple logistic regression, five factors had significant correlation with stress (Tables 4, 5): sex (male: AOR = 0.47, 95% CI = 0.27, 0.83; *p* value = 0.009), watching television for less than an hour (AOR = 0.42, 95% CI = 0.21, 0.82; *p* value = 0.012), going out of the house sometimes (AOR = 0.40, 95% CI = 0.17, 0.98; *p* value = 0.044), any change in the amount of diet (AOR = 2.52, 95% CI = 1.42, 4.48; *p* value = 0.002), having less (AOR = 2.59, 95% CI = 1.37, 4.90; *p* value = 0.003) or more communication with friends than usual (AOR = 2.83, 95% CI = 1.26, 6.34; *p* value = 0.012).

## Discussion

In line with previous studies, we found that females were more prone to mental health problems [15, 18, 24]. The unfortunate fact could explain that women still have to face more challenges than men, especially in South Asian societies [25]. Moreover, girls have to endure certain social norms, constraints and gender discriminations in Bangladesh and other South Asian regions [26, 27]. The constant social pressure is only aggravated by this long-term confinement at home, raising the odds of mental health burdens among females.

Occupation of the mother (public or private services) was associated with anxiety among the participants of this study. In the social context of Bangladesh, women are the homemakers in the family. They take care of the children and share more time with them than the other family members. During this time of crisis, unprecedented to all, the adolescents need more support and reassurance from their parents than usual. Mostly, they look up to their mothers in this regard. Naturally, working mothers do not find as much time for the children as the housewives, contributing to escalating anxiety disorders in adolescents [28]. Equitable distribution of responsibilities between both parents could be a fair solution to this issue. The father should share more of his time with the children to minimize the negative effects of their time away from the mother.

A monthly income of more than 1,00,000 BDT was correlated to lower stress levels in this study. This could be explained by the additional financial demands of this pandemic. Frequent use of disinfectants, hand sanitizer and/or soaps, buying personal protective equipment increased the cost of living in this time of crisis. Moreover, electronic devices and internet connections had to be acquired to continue education online. In the context of these extra expenditures, families with low monthly income faced difficulties providing adequately for their children, which could be responsible for higher levels of perceived stress [29]. This finding was supported by other studies conducted in other Asian countries [15, 30, 31].

Inclination towards online activities makes adolescents physically less active [32]. Confinement at home has exacerbated the situation, compelling teenagers to increase the use of the internet and electronic devices. Researchers have already found a significant association between less physical activity and negative mood [18, 33]. In line with previous findings, we observed fewer chances of depression among the respondents who reported being engaged to minimal work during home quarantine like sports and household chores [34].

Increased screen time has been proved responsible for mental health disturbances among children and adolescents [35]. However, watching television for less than an hour was estimated to be a significant protective factor for stress in the participants. A limited amount of television screen time under parental guidance could be good for the mental health of the teenagers in-home quarantine. Further investigation into this matter is essential.

Most teenagers and young people nowadays engage in internet activities [36]. We discovered that using social networking sites and any form of online leisure (excluding online games) increased the risk of depression and stress among teenagers [37, 38]. Excessive attachment to the internet could disrupt the interpersonal relationships in the family. Moreover, news of widespread infection and deaths and incorrect and misleading information could be creating panic and mental pressure in adolescents and children [39]. However, a study by Magson et al. stated that social media posts play no significant role [40].

We observed lower chances of stress among those who left home sometimes for any purpose, which was a significant finding supported by Francisco et al. [41]. Children and adolescents of developing age need open spaces for their physical and mental growth. We should focus on relieving the adolescents from the imprisonment of their own homes in this pandemic for a regular yet small amount of time.

Changes in eating patterns may show mental health disorders such as depression and anxiety [42, 43]. Losing appetite or overeating are two frequent symptoms of clinical depression [44]. Those who reported an increase or reduction in appetite during quarantine had a higher risk of mental impairment. Communication with friends and peer support could be key in maintaining mental health during the pandemic. We discovered that less communication with friends was linked to higher mental health burdens, consistent with previous research [38, 40]. However, individuals who had more contact with friends than normal during the lockdown were more likely to experience anxiety and tension, a conclusion that we could not understand and that requires further investigation.

Increased levels of stress and anxiety were detected in the adolescents resulting from the unanticipated confinement at home [45]. Continuing education online, despite its drawbacks, could create a sense of purpose, keeping them occupied and intellectually active. A significant association was found between online schoolwork and lower levels of depression during this pandemic [46]. This study also found a significant relationship between online classes and positive mental health. In contrast, Bishwas et al. claimed that distance learning raised depression among the students [47].

The Ministry of Health and Family Welfare in Bangladesh has developed a national strategy to address the diverse health needs of adolescents. The strategy admits the lack of necessary infrastructure to provide psychological support to the country's adolescents. It aims to remove the stigma and ignorance regarding mental health and build the capacity to provide mental health care services integrated with the existing primary healthcare facilities. However, the strategy was first developed in 2017 without considering pandemics [48]. In the light of the recent crisis, further plans of operation should be added to the agenda to address the mental health needs of the adolescents, counting the additional factors in play during home quarantine. It will help figure out the best way to provide mental support and counselling during lockdown.

### Limitations

The study design excluded the adolescents from low socioeconomic status by default as we had to distribute the questionnaire through the internet. For the same reason, we could not formulate a sampling frame and had to depend on our responses after disseminating the Google form, thus following a non-probability sampling technique. Therefore, the generalizability of the study was in question. Keeping the impatient nature of the younger adolescents in mind, we had to keep the survey as short and straightforward as possible, skipping a few variables that we would like to explore otherwise. Finally, the study depended on self-reported answers based on subjective experiences, which may not coordinate with the clinical judgment of mental health professionals.

### Research implications

The goal of this study is to draw the attention of the policymakers to the vulnerable state of adolescent mental health during this pandemic so that they may consider introducing toll-free, specialized psychological help for the adolescents. Surveillance and counselling through apps via the internet could also be implemented to help them adapt better to their circumstances. This research will also help to redesign lockdown protocols in the future by focusing on the factors affecting the mental condition of the younger generations and allowing some room to relieve the burdens on their minds.

## Conclusion

The study's key findings show that life in quarantine has harmed adolescents' mental health at the most vulnerable stage of their lives. A notable portion of this age group is affected by depression, anxiety and/or stress due to various personal, academic, social and familial factors owed to the pandemic. Despite being a fundamental issue, adolescent mental health has not yet received the spotlight it deserves in Bangladesh. This study strongly recommends that we do not forget to pay more attention to adolescents' mental health while combating the coronavirus pandemic.

## Data Availability

The data can be provided on a valid request to Dr. Mohammad Delwer Hossain Hawlader, Dept. of Public Health, North South University, Dhaka-1229, Bangladesh, Email: mohammad.hawlader@northsouth.edu.

## Abbreviations

DASS-21: Depression, Anxiety and Stress Scale-21
WHO: World Health Organization
OR: Odds ratio
AOR: Adjusted odds ratio
COVID-19: Coronavirus disease 2019
SARS-CoV-2: Severe acute respiratory syndrome coronavirus 2
